# Bis(acetato-κ^2^ *O*,*O*′)(4,4′-dimethyl-2,2′-bipyridine-κ^2^ *N*,*N*′)­zinc

**DOI:** 10.1107/S1600536812042699

**Published:** 2012-10-20

**Authors:** Miguel A. Harvey, Sebastian A. Suarez, Andres Ibañez, Fabio Doctorovich, Ricardo Baggio

**Affiliations:** a Universidad Nacional de la Patagonia S.J.B. and Centro Nacional Patagonico, CONICET, Bvd. Alte. Brown 3700, 9120 Puerto Madryn, Chubut, Argentina; b Departamento de Química Inorgánica, Analítica y Química, Física/INQUIMAE-CONICET, Facultad de Ciencias Exactas y Naturales, Universidad de Buenos Aires, Argentina; c Laboratorio de Cristalografía, Difracción de Rayos-X, Facultad de Ciencias Físicas y Matemáticas, Universidad de Chile. Av. Blanco Encalada 2008, Santiago, Chile; d Gerencia de Investigación y Aplicaciones, Centro Atómico Constituyentes, Comisión Nacional de Energía Atómica, Buenos Aires, Argentina

## Abstract

The mol­ecular structure of the title compound, [Zn(CH_3_COO)_2_(C_12_H_12_N_2_)], consists of isolated mol­ecules bis­ected by a twofold rotation axis which goes through the Zn^II^ cation and halves the organic base through the central C—C bond. The Zn^II^ ion is coordinated by two N atoms from one mol­ecule of the aromatic base and four O atoms from two bidentate, symmetry-related acetate anions, which coordinate asym­metrically [Zn—O distances of 2.058 (2) and 2.362 (3) Å], while the two Zn—N bond distances are equal as imposed by symmetry [2.079 (2) Å]. The crystal structure is supported by a number of weak C—H⋯O inter­actions and C—H⋯π contacts, with no π–π inter­actions present, mainly hindered by the substituent methyl groups and the relative mol­ecular orientation. The result is a three-dimensional structure in which each mol­ecule is linked to eight different neighbors.

## Related literature

For properties of polypyridyl compounds, see: Steed & Atwood (2009 ▶). For related structures, see: Barquín *et al.* (2010 ▶). For details of the vectorial bond–valence model, see Harvey *et al.* (2006 ▶).
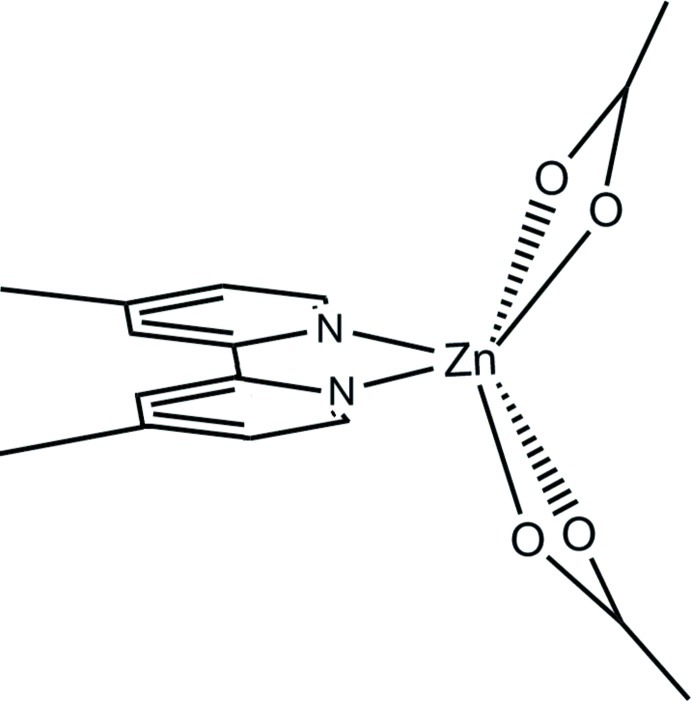


## Experimental

### 

#### Crystal data

[Zn(C_2_H_3_O_2_)_2_(C_12_H_12_N_2_)]
*M* *_r_* = 367.71
Orthorhombic, 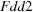

*a* = 14.4779 (5) Å
*b* = 28.5700 (15) Å
*c* = 8.0854 (3) Å
*V* = 3344.4 (2) Å^3^
*Z* = 8
Mo *K*α radiation
μ = 1.49 mm^−1^
*T* = 295 K
0.3 × 0.3 × 0.2 mm

#### Data collection

Oxford Diffraction Gemini CCD S Ultra diffractometer
Absorption correction: multi-scan (*CrysAlis PRO*; Oxford Diffraction, 2009 ▶) *T*_min_ = 0.65, *T*_max_ = 0.75
3945 measured reflections
1563 independent reflections
1481 reflections with *I* > 2σ(*I*)
*R*_int_ = 0.015

#### Refinement

*R*[*F* ^2^ > 2σ(*F* ^2^)] = 0.026
*wR*(*F* ^2^) = 0.068
*S* = 1.09
1563 reflections
107 parameters
1 restraint
H-atom parameters constrained
Δρ_max_ = 0.28 e Å^−3^
Δρ_min_ = −0.26 e Å^−3^
Absolute structure: Flack (1983 ▶), 374 Friedel pairs
Flack parameter: 0.010 (16)

### 

Data collection: *CrysAlis PRO* (Oxford Diffraction, 2009 ▶); cell refinement: *CrysAlis PRO*; data reduction: *CrysAlis PRO*; program(s) used to solve structure: *SHELXS97* (Sheldrick, 2008 ▶); program(s) used to refine structure: *SHELXL97* (Sheldrick, 2008 ▶); molecular graphics: *SHELXTL* (Sheldrick, 2008 ▶); software used to prepare material for publication: *SHELXL97* and *PLATON* (Spek, 2009) ▶.

## Supplementary Material

Crystal structure: contains datablock(s) global, I. DOI: 10.1107/S1600536812042699/br2212sup1.cif

Structure factors: contains datablock(s) I. DOI: 10.1107/S1600536812042699/br2212Isup2.hkl

Additional supplementary materials:  crystallographic information; 3D view; checkCIF report

## Figures and Tables

**Table 1:** Hydrogen-bond geometry (Å, °) *Cg*1 and *Cg*2 are the centroids of the Zn1,O1,C7,O2 and N1,C1–C5 rings, respectively.

*D*—H⋯*A*	*D*—H	H⋯*A*	*D*⋯*A*	*D*—H⋯*A*
C2—H2⋯O1^i^	0.93	2.53	3.354 (4)	147
C6—H6*A*⋯O2^ii^	0.96	2.56	3.438 (4)	153
C4—H4⋯*Cg*1^iii^	0.93	2.99	3.874 (4)	160
C4—H4⋯*Cg*1^ii^	0.93	2.96	3.766 (4)	145
C8—H8*B*⋯*Cg*2^iv^	0.96	2.96	3.804 (4)	147

Symmetry codes: (i) 
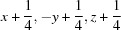
; (ii) 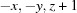; (iii) 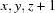; (iv) 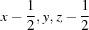.

**Fig. 1.**
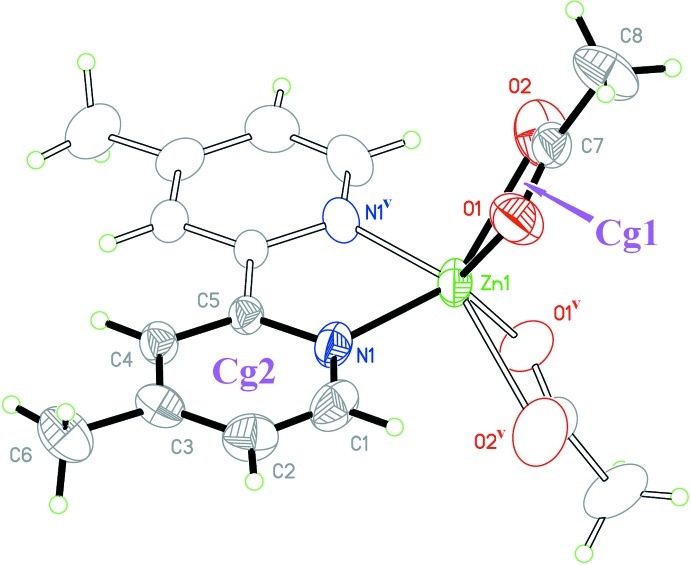
Ellipsoid plot of (I), drawn with displacement factors at a 40% probability level. In full(empty) ellipsods and bonds, the independent(symmetry related) part of the structure. Symmetry code: (v): -x, -y, z

**Fig. 2.**
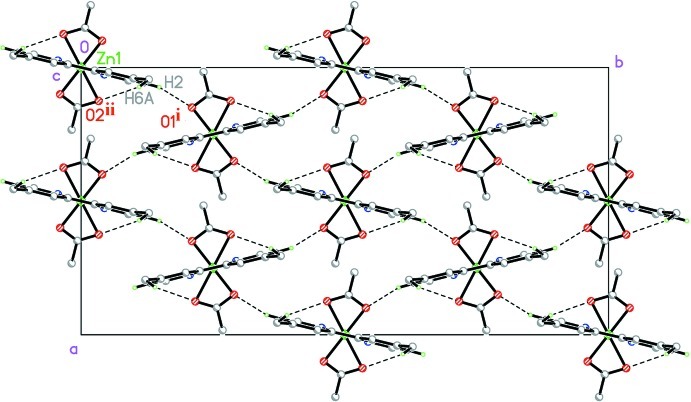
Packing view projected down c. Only the C—H···O interactions have been drawn (in broken lines). H atoms not invovled in these interactions have been omitted, for clarity. Symmetry codes: (i) x + 1/4, -y + 1/4, z + 1/4; (ii) -x, -y, z + 1.

## supplementary crystallographic information

### Comment

Polypyridil compounds and some of their derivatives have shown to be fruitful ligands in supramolecular photochemistry, due to the capability of its extended *π*- systems to absorb light. They can act as light harvesters as much as to relax photoexcited metal centres *via* MLCT to the ligand-centred *π**_L_ orbital; some interesting examples can be found in Steed & Atwood, 2009. In particular, in the case of 4,4'-dimethyl-2,2'-bipyridine (dmbp), the presence of the methyl groups in the aromatic ligand can additionally influence the structural behavior when binding to a metal centre. We present in what follows the crystal and molecular structure of the title compound, C_16_H_18_N_2_O_4_Zn, consisting of isolated Zn(dmbp)(*ac*)_2_, molecules (*ac* = acetate) bisected by a twofold axis which goes through the Zn(II) cation and halves the organic base through the central C—C bond.

The Zn(II) ion is coordinated by two nitrogen atoms from one molecule of the aromatic base and four oxygen atoms from two bidentate, symmetry related acetate anions (Fig. 1). A very similar compound, with Cu(II) as its central cation has been reported in Barquín *et al.*, 2010. Donor atoms in the title compound can not fit in any regular polyhedron, but the three chelate ligands fulfill the *vector bond valence* postulate of the *Vectorial Bond-Valence Model* (for details on the theory see Harvey *et al.*, 2006). The three ligand vectors, as defined therein, lay in a planar trigonal geometry with a sum of angles equal to 359.6 (2)° (ideal: 360°) and a resultant vector modulus of 0.03 v.u. (Ideal: 0.00 v.u.).

Both acetate anions coordinate asymmetrically (Zn—O distances 2.058 (2) and 2.362 (3) Å), while the two Zn—N bond distances are equal (2.079 (2) Å) as imposed by symmetry.

The crystal structure is supported by a number of weak C—H···O interactions (Table 1, entries 1,2) and C—H···π contacts (Table 1, entries 3 to 5). In spite of the presence of aromatic rings there are no π-π interactions in the structure, mainly hindered by the substituent methyl groups and the relative molecular orientation.

The overall effect of these weak interactions, uniformly distributed in space, is the formation of a three-dimensional structure where each molecule is linked to eight different neighbors. Fig. 2 presents a highly simplified packing view projected down c, where only the C—H···O bonds have been drawn, for clarity, and where the complex linkage can be envisaged.

### Experimental

The title compound was obtained as an unexpected byproduct in an attempt to synthesize a Zn tetrathionate complex with the aromatic base. Solid Zn acetate dihydrate, 4,4'-Dimethylbipyridine and potassium tetrathionate, 0.050 mmol of each, were added to 5 ml of dimethylformamide. On standing, colorless blocks of the title compound could be extracted for diffraction experiments.

### Refinement

All H atoms were confirmed in a difference map, further idealized and allowed to ride, with displacement parameters taken as *U*_iso_(H) = *X* × *U*_eq_(C) [(C—H) methyl = 0.96 A°, *X* = 1.5; (C—H) arom = 0.93 A°, *X* = 1.2] (CH_3_ groups were also free to rotate as well).

### Crystal data

**Table:**

[Zn(C_2_H_3_O_2_)_2_(C_12_H_12_N_2_)]	*F*(000) = 1520
*M**_r_* = 367.71	*D*_x_ = 1.461 Mg m^−^^3^
Orthorhombic, *F**d**d*2	Mo *K*α radiation, λ = 0.71073 Å
Hall symbol: F 2 -2d	Cell parameters from 1985 reflections
*a* = 14.4779 (5) Å	θ = 3.6–28.9°
*b* = 28.5700 (15) Å	µ = 1.49 mm^−^^1^
*c* = 8.0854 (3) Å	*T* = 295 K
*V* = 3344.4 (2) Å^3^	Prism, white
*Z* = 8	0.3 × 0.3 × 0.2 mm

### Data collection

**Table:**

Oxford Diffraction Gemini CCD S Ultra diffractometer	1481 reflections with *I* > 2σ(*I*)
Graphite monochromator	*R*_int_ = 0.015
ω scans, thick slices	θ_max_ = 29.0°, θ_min_ = 3.6°
Absorption correction: multi-scan (*CrysAlis PRO*; Oxford Diffraction, 2009)	*h* = −17→18
*T*_min_ = 0.65, *T*_max_ = 0.75	*k* = −37→17
3945 measured reflections	*l* = −10→5
1563 independent reflections	

### Refinement

**Table:**

Refinement on *F*^2^	Secondary atom site location: difference Fourier map
Least-squares matrix: full	Hydrogen site location: difference Fourier map
*R*[*F*^2^ > 2σ(*F*^2^)] = 0.026	H-atom parameters constrained
*wR*(*F*^2^) = 0.068	*w* = 1/[σ^2^(*F*_o_^2^) + (0.0402*P*)^2^ + 1.025*P*] where *P* = (*F*_o_^2^ + 2*F*_c_^2^)/3
*S* = 1.09	(Δ/σ)_max_ < 0.001
1563 reflections	Δρ_max_ = 0.28 e Å^−^^3^
107 parameters	Δρ_min_ = −0.26 e Å^−^^3^
1 restraint	Absolute structure: Flack (1983), 374 Friedel pairs
Primary atom site location: structure-invariant direct methods	Flack parameter: 0.010 (16)

### Special details

**Table:**

Geometry. All e.s.d.'s (except the e.s.d. in the dihedral angle between two l.s. planes) are estimated using the full covariance matrix. The cell e.s.d.'s are taken into account individually in the estimation of e.s.d.'s in distances, angles and torsion angles; correlations between e.s.d.'s in cell parameters are only used when they are defined by crystal symmetry. An approximate (isotropic) treatment of cell e.s.d.'s is used for estimating e.s.d.'s involving l.s. planes.
Refinement. Refinement of *F*^2^ against ALL reflections. The weighted *R*-factor *wR* and goodness of fit *S* are based on *F*^2^, conventional *R*-factors *R* are based on *F*, with *F* set to zero for negative *F*^2^. The threshold expression of *F*^2^ > σ(*F*^2^) is used only for calculating *R*-factors(gt) *etc*. and is not relevant to the choice of reflections for refinement. *R*-factors based on *F*^2^ are statistically about twice as large as those based on *F*, and *R*- factors based on ALL data will be even larger.

### Fractional atomic coordinates and isotropic or equivalent isotropic displacement parameters (Å^2^)

**Table:**

	*x*	*y*	*z*	*U*_iso_*/*U*_eq_	
Zn1	0	0	0.08552 (5)	0.04845 (13)	
O1	−0.10026 (14)	0.03972 (8)	−0.0279 (3)	0.0745 (6)	
N1	0.02563 (14)	0.04418 (7)	0.2846 (2)	0.0456 (4)	
O2	−0.12535 (18)	−0.03328 (9)	−0.0599 (3)	0.0843 (7)	
C4	0.01794 (12)	0.05180 (7)	0.5779 (5)	0.0421 (5)	
H4	0.0071	0.038	0.6803	0.051*	
C7	−0.14861 (19)	0.00731 (10)	−0.0837 (4)	0.0548 (7)	
C5	0.01168 (12)	0.02544 (8)	0.4354 (3)	0.0361 (4)	
C8	−0.2331 (2)	0.01806 (14)	−0.1800 (9)	0.0900 (14)	
H8B	−0.2777	0.0328	−0.1093	0.135*	
H8C	−0.2584	−0.0104	−0.2238	0.135*	
H8A	−0.2178	0.0388	−0.2694	0.135*	
C3	0.04052 (15)	0.09917 (8)	0.5683 (4)	0.0502 (5)	
C1	0.0483 (2)	0.08971 (10)	0.2769 (4)	0.0604 (7)	
H1	0.0586	0.103	0.1735	0.073*	
C2	0.05697 (19)	0.11722 (9)	0.4128 (4)	0.0607 (7)	
H2	0.0741	0.1484	0.401	0.073*	
C6	0.0449 (2)	0.12828 (10)	0.7225 (4)	0.0719 (9)	
H6A	0.0721	0.1103	0.8101	0.108*	
H6C	−0.0164	0.1376	0.7538	0.108*	
H6B	0.0818	0.1556	0.7024	0.108*	

### Atomic displacement parameters (Å^2^)

**Table:**

	*U*^11^	*U*^22^	*U*^33^	*U*^12^	*U*^13^	*U*^23^
Zn1	0.04289 (17)	0.0737 (3)	0.02871 (17)	0.01368 (17)	0	0
O1	0.0622 (11)	0.0907 (14)	0.0706 (15)	−0.0028 (10)	−0.0158 (11)	−0.0093 (12)
N1	0.0478 (10)	0.0549 (11)	0.0342 (11)	0.0047 (8)	0.0032 (8)	0.0080 (8)
O2	0.0909 (16)	0.0871 (15)	0.0749 (17)	0.0313 (12)	−0.0146 (13)	−0.0022 (13)
C4	0.0424 (11)	0.0480 (10)	0.0361 (11)	0.0034 (8)	−0.0042 (16)	0.0013 (12)
C7	0.0399 (12)	0.0850 (19)	0.0393 (14)	0.0085 (11)	0.0013 (10)	−0.0056 (13)
C5	0.0329 (9)	0.0459 (12)	0.0296 (11)	0.0036 (7)	−0.0017 (8)	0.0031 (9)
C8	0.061 (2)	0.109 (3)	0.100 (4)	0.0012 (17)	−0.037 (3)	0.017 (3)
C3	0.0452 (10)	0.0462 (11)	0.0593 (16)	0.0006 (9)	−0.0095 (12)	−0.0025 (12)
C1	0.0654 (15)	0.0602 (15)	0.0557 (18)	−0.0014 (12)	0.0060 (12)	0.0240 (13)
C2	0.0621 (16)	0.0440 (12)	0.076 (2)	−0.0032 (11)	−0.0005 (14)	0.0087 (14)
C6	0.080 (2)	0.0559 (15)	0.079 (2)	−0.0007 (14)	−0.0190 (17)	−0.0158 (16)

### Geometric parameters (Å, º)

**Table:**

Zn1—O1^i^	2.058 (2)	C4—H4	0.93
Zn1—O1	2.058 (2)	C7—C8	1.482 (5)
Zn1—N1	2.079 (2)	C5—C5^i^	1.493 (4)
Zn1—N1^i^	2.079 (2)	C8—H8B	0.96
Zn1—O2	2.362 (3)	C8—H8C	0.96
Zn1—O2^i^	2.362 (3)	C8—H8A	0.96
Zn1—C7	2.558 (3)	C3—C2	1.380 (4)
Zn1—C7^i^	2.558 (3)	C3—C6	1.500 (4)
O1—C7	1.246 (3)	C1—C2	1.357 (4)
N1—C1	1.343 (3)	C1—H1	0.93
N1—C5	1.347 (3)	C2—H2	0.93
O2—C7	1.223 (3)	C6—H6A	0.96
C4—C5	1.380 (4)	C6—H6C	0.96
C4—C3	1.394 (3)	C6—H6B	0.96
			
O1^i^—Zn1—O1	127.09 (15)	C5—C4—C3	119.9 (3)
O1^i^—Zn1—N1	123.63 (9)	C5—C4—H4	120
O1—Zn1—N1	97.82 (8)	C3—C4—H4	120
O1^i^—Zn1—N1^i^	97.82 (8)	O2—C7—O1	119.6 (3)
O1—Zn1—N1^i^	123.63 (9)	O2—C7—C8	120.4 (3)
N1—Zn1—N1^i^	78.52 (11)	O1—C7—C8	120.0 (3)
O1^i^—Zn1—O2	95.63 (10)	O2—C7—Zn1	66.85 (17)
O1—Zn1—O2	57.21 (9)	O1—C7—Zn1	52.72 (15)
N1—Zn1—O2	140.08 (8)	C8—C7—Zn1	172.7 (2)
N1^i^—Zn1—O2	90.22 (9)	N1—C5—C4	122.0 (2)
O1^i^—Zn1—O2^i^	57.21 (9)	N1—C5—C5^i^	114.92 (13)
O1—Zn1—O2^i^	95.63 (10)	C4—C5—C5^i^	123.13 (15)
N1—Zn1—O2^i^	90.22 (9)	C7—C8—H8B	109.5
N1^i^—Zn1—O2^i^	140.08 (8)	C7—C8—H8C	109.5
O2—Zn1—O2^i^	120.30 (15)	H8B—C8—H8C	109.5
O1^i^—Zn1—C7	113.57 (9)	C7—C8—H8A	109.5
O1—Zn1—C7	28.79 (8)	H8B—C8—H8A	109.5
N1—Zn1—C7	120.97 (8)	H8C—C8—H8A	109.5
N1^i^—Zn1—C7	108.27 (9)	C2—C3—C4	117.0 (3)
O2—Zn1—C7	28.42 (8)	C2—C3—C6	122.9 (2)
O2^i^—Zn1—C7	110.31 (10)	C4—C3—C6	120.1 (3)
O1^i^—Zn1—C7^i^	28.79 (8)	N1—C1—C2	123.1 (2)
O1—Zn1—C7^i^	113.57 (9)	N1—C1—H1	118.5
N1—Zn1—C7^i^	108.27 (9)	C2—C1—H1	118.5
N1^i^—Zn1—C7^i^	120.97 (8)	C1—C2—C3	120.4 (2)
O2—Zn1—C7^i^	110.31 (10)	C1—C2—H2	119.8
O2^i^—Zn1—C7^i^	28.42 (8)	C3—C2—H2	119.8
C7—Zn1—C7^i^	115.34 (13)	C3—C6—H6A	109.5
C7—O1—Zn1	98.50 (18)	C3—C6—H6C	109.5
C1—N1—C5	117.6 (2)	H6A—C6—H6C	109.5
C1—N1—Zn1	126.56 (18)	C3—C6—H6B	109.5
C5—N1—Zn1	115.64 (15)	H6A—C6—H6B	109.5
C7—O2—Zn1	84.73 (19)	H6C—C6—H6B	109.5

Symmetry code: (i) −*x*, −*y*, *z*.

### Hydrogen-bond geometry (Å, º)

Cg1 and Cg2 are the centroids of the Zn1,O1,C7,O2 and N1,C1–C5 rings, respectively.

**Table:**

*D*—H···*A*	*D*—H	H···*A*	*D*···*A*	*D*—H···*A*
C2—H2···O1^ii^	0.93	2.53	3.354 (4)	147
C6—H6*A*···O2^iii^	0.96	2.56	3.438 (4)	153
C4—H4···*Cg*1^iv^	0.93	2.99	3.874 (4)	160
C4—H4···*Cg*1^iii^	0.93	2.96	3.766 (4)	145
C8—H8*B*···*Cg*2^v^	0.96	2.96	3.804 (4)	147

Symmetry codes: (ii) *x*+1/4, −*y*+1/4, *z*+1/4; (iii) −*x*, −*y*, *z*+1; (iv) *x*, *y*, *z*+1; (v) *x*−1/2, *y*, *z*−1/2.
